# Gout in Its Clinical Aspects

**Published:** 1885-09

**Authors:** 


### Notes on Books.

*Gout in its Clinical Aspects.* By J. MORTIMER GRANVILLE, M.D. London: J. & A. CHURCHILL. 1885.

*Gout, and its Relations to Diseases of the Liver and Kidneys.* By ROBSON ROOSE, M.D. London: H. K. LEWIS. 1885.

These two literary contributions, founded on the personal experience of their respective authors, lay no claim to be exhaustive treatises, or to supplant the older monograph of Professor Garrod. Both are clinical manuals, which, whilst they give a general outline of the subject culled from various sources, and direct attention to certain modern theories as to the origin and cause of the disease, are records of personal observation and individual thought. The former has much which will be new to the reader, inasmuch as the experience of the author differs in some points from what is commonly accepted; and the fertility of therapeutic resource, together with the unbounded belief in the efficacy of all the drugs he employs, are quite refreshing features in this too-unbelieving age. The latter book aims to prove that functional disorder of the liver underlies the majority of gouty manifestations, and that the kidneys are only secondarily implicated; it follows that the object of treatment should be not merely the neutralisation of the *materies morbi* by means of alkalies, but, in an especial manner, the restoration of the hepatic functions.

As regards Dr. Granville's book, there is much that is new; but opinions will be likely to differ as to whether all that is new is true. For instance, page 23: "The idea that children are not affected with gout is manifestly fallacious; and I cannot help thinking that the disorder is of quite as frequent occurrence among females as among males."

Again, page 157: "I think I have seen acute gout in infants even during the period of suckling. I have no doubt as to the gouty character of inflammatory swellings of the joints occurring, with distinct pyrexia, and subsequent peeling of the skin of the swollen limb, as early as the third year." "Gouty deafness in children is a familiar affection, the deposits being visible through the out-layers of the membrana tympani. Concretions are also to be found in the cartilages of the ears of children: some of the so-called 'cretaceous' accumulations under the prepuce are gouty, and I believe gout is responsible for not a few of the attacks of vaginitis or vulvitis, which are occasionally attributed to the irritation set up by worms."

Again, page 139: "The walls of the vessels of the encephalon are not unfrequently the seats of gouty deposit, and liable to give way suddenly."

Page 41: "Cases are on record in which the skin has been covered every morning with what is popularly called 'chalk,' giving the patient the appearance of having been dusted with white powder."

Page 149: "Gouty inflammation of, and deposits in, the larynx, with deposits of water in or beneath the vocal cords, are easily recognised."

Page 148: "I am confident that specially gouty inflammations attack other nerves, and why not the optic? I think that optic neuritis may be of gouty origin."

Page 46: "I have certainly found well-formed crystals of uric acid in the blood in the vessels of the brain after death, in cases of gouty inflammation of that organ, or, more accurately speaking, cerebral meningitis occurring in irregular gout."

Dr. Roose's account of treatment comprises a description of the various methods—dietetic, medicinal, and hygienic—which are commonly practised, and of which the utility has been abundantly proved. There is little to comment upon or to call in question; but it is a well-written and interesting account of what the careful and non-speculative physician may accomplish in the different phases of the disease. On the other hand, Dr. Granville's account includes much which is contrary to general practice and experience, much which is speculative, and much which is at least suggestive of hasty inductions from deficient premises. The author must, however, be allowed to speak for himself as to the utility of his armamentarium, which includes a great variety of drugs, each having its definite function, and which, in the author's remarkably fortunate experience, it rarely fails to perform, even when in conjunction with numerous others in elaborate combination. Numerous formulæ are appended, amounting to no fewer than one hundred and fifty-two, each of which has its special uses; and a perusal of which cannot fail to be of service to the busy practitioner who has no time to investigate the action of new drugs, but will be glad to utilise those which have been found to be of such service by so painstaking and thoughtful an observer as Dr. Mortimer Granville.

